# Reconstructing a COVID-19 outbreak within a religious group using social network analysis simulation in Korea

**DOI:** 10.4178/epih.e2021068

**Published:** 2021-09-16

**Authors:** Namje Kim, Su Jin Kang, Sangwoo Tak

**Affiliations:** 1 Department of Economics, Seoul National University, Seoul, Korea; 2 Institute of Research in Finance and Economics, Seoul National University, Seoul, Korea; 3 Institute of Health and Environment, Seoul National University, Seoul, Korea

**Keywords:** COVID-19, Social network analysis, Physical distancing

## Abstract

**OBJECTIVES:**

We reconstructed a coronavirus disease 2019 (COVID-19) outbreak to examine how a large cluster at a church setting spread before being detected and estimate the potential effectiveness of complying with mask-wearing guidelines recommended by the government.

**METHODS:**

A mathematical model with a social network analysis (SNA) approach was used to simulate a COVID-19 outbreak. A discrete-time stochastic simulation model was used to simulate the spread of COVID-19 within the Sarang Jeil church. A counterfactual experiment using a calibrated baseline model was conducted to examine the potential benefits of complying with a mask-wearing policy.

**RESULTS:**

Simulations estimated a mask-wearing ratio of 67% at the time of the outbreak, which yielded 953.8 (95% confidence interval [CI], 937.3 to 970.4) cases and was most consistent with the confirmed data. The counterfactual experiment with 95% mask-wearing estimated an average of 45.6 (95% CI, 43.4 to 47.9) cases with a standard deviation of 20.1. The result indicated that if the church followed government mask-wearing guidelines properly, the outbreak might have been one-twentieth the size.

**CONCLUSIONS:**

SNA is an effective tool for monitoring and controlling outbreaks of COVID-19 and other infectious diseases. Although our results are based on simulations and are thus limited, the precautionary implications of social distancing and mask-wearing are still relevant. Since person-to-person contacts and interactions are unavoidable in social and economic life, it may be beneficial to develop precise measures and guidelines for particular organizations or places that are susceptible to cluster outbreaks.

## INTRODUCTION

The ongoing coronavirus disease 2019 (COVID-19) pandemic has resulted in more than 166 million confirmed cases and more than 3 million deaths worldwide as of the time of this study [1]. In Korea, more than 136,000 confirmed cases and 1,934 deaths have been reported as of May 24, 2021 [2]. Korea has had several large-scale COVID-19 outbreaks [2]. The first wave emerged between February and March 2020 among members of a religious group located in Daegu that was linked to more than 5,200 cases. The second wave began to emerge in mid-August 2020 among members of the Sarang Jeil (SJ) church in Seoul. Media coverage widely reported that the leader and members of the SJ church did not follow government guidelines related to COVID-19 prevention, such as social distancing and mask-wearing, resulting in a total of 959 confirmed case by August 28, 2020 (and 1,163 cases by September 7, 2020) [3].

Korea has conducted extensive contact tracing, testing, and isolation measures since the beginning of the COVID-19 outbreak. Large-scale contact tracing helps identify pre-symptomatic and asymptomatic cases and plays a critical role in successfully controlling disease spread [4-7]. Unfortunately, since every contact must be traced, resulting in the identification of a high number of positive cases over a very short period of time, extensive contact tracing can lead to a sharp increase in the number of cases to a peak of the epidemic curve, and transmission parameters based on the number of daily confirmed cases, such as the basic reproductive number, can lead to overestimation [8]. Since massive COVID-19 outbreaks have been mostly triggered by local clusters of cases that spread unrecognized, it is important to examine micro-level dynamics to recommend effective intervention measures and ultimately prevent future potential large-scale outbreaks. To understand how this example of local transmission in a church setting continued before being detected, we developed a stochastic network simulation model in an attempt to reconstruct the COVID-19 outbreak at the SJ church using social network analysis (SNA). We also estimated the potential effectiveness of complying with mask-wearing guidelines recommended by the government.

## MATERIALS AND METHODS

### Simulation model

To reconstruct the outbreak events and compare the effectiveness of intervention measures, we constructed a discrete-time stochastic simulation model of the spread of COVID-19 within a structured environment. SNA was used to construct an environment similar to that of the SJ church. In general, SNA is used to study the behaviors of individuals at the micro level, the patterns of relationships at the macro level, and the interactions between the two [9].

A typical social network representation has nodes for people connected by edges to represent 1 or more relationships between them [10]. SNA has been widely used in the field of epidemiology [11-13]. Using SNA, we generated a worship hall (sanctuary) and designed a simulation model that included daily church events. The parameters were calibrated to match the data from confirmed cases among the members of the SJ church. Individual nodes represented each person, while edges captured the interactions between them (i.e., vectors of infection transmission), thereby simulating random contacts between members of the community in a virtual space. To reflect the heterogeneous nature of the infection, we ran simulations based on probabilities of infection, which depended on the degree of contact with infected people. The degree of contact was based on the physical distance from the infected person, whether masks were worn, and the number of infected people sitting nearby. This enables good heterogeneity for the likelihood of viral transmission. We used Python 3.6.4 (Python Software Foundation, Wilmington, DE, USA) and the following packages in our study: (1) “NumPy” to build the simulation model, (2) “NetworkX” to generate the network structure, (3) “Matplotlib” and “seaborn” to print out plots and graphs, and (4) “Pupyter notebook” to edit the program.

### Model description

To conduct the simulation, we created a virtual environment similar to the main hall of the SJ church. The number of seats in the main hall was estimated based on multiple media sources. The structure of the main hall was separated into 4 sections. Each section was connected by a passage through which people could move. The left and right sections had short pews that seated about 4 people, and the 2 sections in the center had long pews that seated about 5 people. We estimated that 20 rows to 22 rows were placed on the first floor of the hall, and 3 rows to 4 rows were placed on the second floor. In addition, Sunday worship attendees were estimated to exceed the main hall capacity, as chairs and a large screen were placed outdoors next to the church for Sunday worship. Therefore, we estimated the capacity of the main hall to be 540 seats.

### Subjects

The subjects were grouped into 3 categories: pastors and church staff members, registered church members, and non-registered visitors. In total, 25 pastors and staff members, including the head pastor, were listed on the SJ church website (https://sarangjeil.com/). We assumed that these pastors and staff members visited the church every day and attended daily events at random when they were not required to attend. We also assumed that all pastors attended the main worship services on Sundays. The daily events of the church are described in the next section. Lastly, we assumed that 2,222 registered members and 3,600 non-registered visitors attended services at the church during the simulation period [14].

### Daily events at the Sarang Jeil church

The SJ church held several worship services throughout each week: daily morning prayers, weekday worship services on Wednesdays, all-night worship services on Fridays, small group gatherings on Saturdays, and 3 main worship services on Sundays. Daily morning prayers are held in the early morning hours on weekdays. In our model, the participants in daily worship service were assumed to be 10-30 random registered members and 5 random pastors and staff members. Thus, the possible number of participants was 15-35 people. For weekday worship services, we assumed that 50% of the main hall would be filled. Therefore, it was assumed that 150-200 registered members, 0-50 outside visitors, and all pastors participated in weekday worship services, and the possible number of participants was 175-275. On Saturdays, 8 small group gatherings were held, and each group was assumed to have 20-30 people. To simplify the model, we assumed that all gatherings were held in the main hall with 180-220 registered members and all pastors present. Thus, the possible number of participants was 205-245. For the main worship services on Sundays, it was assumed that the main hall was filled every time and all pastors attended, with two-thirds of the remaining seats being filled by registered members and one-third by outside visitors. Finally, we assumed that participants who already attended a Sunday service would not attend the other services. Daily church events are summarized in Table 1.

### Baseline model

#### Simulation process and calibration

The simulation was conducted based on the period from July 27 to August 28, 2020, and the types of events simulated were determined based on the day of the week. The construction of participants at each event was as follows. First, the number of participants in each group (i.e., pastors, members, or visitors) was randomly assigned using uniform distribution based on the assumptions outlined in the previous section. Next, the participants from each group were selected via a lottery based on the designated number of people for each event. Once all participants were selected, seats in the main hall were chosen for each participant via a random lottery. Once all seats were filled, each participant began to make contact with other people within his or her contact distance during the event. Due to the limited data conditions and the need to simplify the assumptions, an a priori model was formulated for this study. First, we normalized the distance of 2 adjacent seats to 1 to indicate the shortest possible distance between any 2 participants. Next, participants were assigned to different contact distances based on their roles. The contact distance of registered members and visitors was assumed to be 3, which is approximately 1 m, equal to the length of a short pew. Pastors and church staff members, however, tend to move and make contact with others during church events (e.g., greeting participants before and after the service, handing out church bulletins, guiding seating, and checking the audio equipment). Their contact distance was set to 6 accordingly. We estimated that each staff member contacted 60-80 people on average during main worship services on Sundays.

We developed a probability of infection in a worst-case setting, which was the probability of an individual being infected after sitting adjacent to an infected person for 1 hour with neither person wearing a mask and denoted it as *P*_1_. One recent study on COVID-19 [15] estimated the transmission probability of dental healthcare workers at 99.4% under unfavorable environmental conditions. The SJ church shared a similar environment to that of the study of a dental clinic in terms of face-to-face distance and 1 hour of exposure time. However, the air ventilation was assumed to be slightly better in the SJ church since there were windows on the right side of the main hall opened during the services. This means that the main hall was crowded but not completely enclosed. In addition, the direction of face-to-face contact between individuals also differed from the dental clinic setting. During church events, every participant faces the same direction. Given these factors, we assumed a relatively moderate transmission probability at 80% for *P*_1_. Hu et al. [16] estimated the probability of infection on a high-speed train and found a significant negative correlation between the probability of infection and the distance between seats of an infected person. Therefore, we defined the probability of infection (*P_d_*) as a function of the distance between 2 people (*d*):


$$P_{d}=\frac{P_{1}}{d}$$

An important feature of this model was mask-wearing. COVID-19 is transmitted primarily via respiratory droplets, and maskwearing has been confirmed to be one of the current most effective measures to mitigate the airborne transmission of COVID-19. Recently, Ueki et al. [17] conducted an experiment on COVID-19 transmission using a mannequin at the University of Tokyo. The results showed that viral transmission decreased by 95.0% to 99.6% when an uninfected person was exposed to an infected person for 20 minutes within a 50 cm distance and only the infected person wore an N95 respirator. In contrast, viral transmission decreased by 57% to 79% when only the non-infected person wore an N95 respirator. Another study conducted in Korea compared the adequate protection rate of N95 and KF94 masks using 2 different metrics: fit factor and leakage rate [18]. The researchers found that N95 respirators had a significantly higher adequate protection rate (N95: 48.7% vs. KF94: 1.1% by fit factor and N95: 42% vs. KF94: 2.8% by leakage rate). Given that the distance between adjacent seats was shorter than 50 cm, the exposure time was much longer (1 hour), and the protection rate of KF94 masks is lower than that of N95 masks, we assigned a 90% protection rate when only the infected person wore a mask and a 60% protection rate when only the non-infected person wore a mask, which was lower than the mask protection rate suggested by Ueki et al. [17]. Lastly, a 100% protection rate was assumed if both individuals wore masks (Table 2).

An increase in the number of infected people sitting nearby also affects the probability of infection. For example, if a non-infected person sits adjacent to 1 infected person, the probability of infection is *P_d_*. If a non-infected person sits adjacent to 2 infected people, the probability of infection becomes 1−(1−*P_d_*)^2^. Therefore, the probability of infection has great heterogeneity since it is determined by (1) distance from the infected person, (2) maskwearing conditions, and (3) the number of infected people sitting nearby.

Once a person becomes infected, they enter the incubation period. Based on a study conducted by Li et al. [19], we used an average incubation period of 5.2 days. Another study by Min et al. [20] reported that individuals typically develop a COVID-19 infection 1 day before symptoms are present; therefore, we used an average latent period of 4.2 days. After the latent period, the patient was considered contagious. While Li et al. [19] reported that the average period from the onset of symptoms to entering quarantine was 12.5 days before January 1 and 9.1 days between January 1 and 11 in the early phase of the pandemic in Wuhan, China in 2020, Min et al. [20] found an average of 4.8 days during the first wave of the pandemic in Korea. This gap is not negligible. An efficient tracking system and increased public awareness can shorten this interval. Therefore, we adopted a middle value between the 2 studies, estimating an average period of onset to quarantine at 7.9 days and an infectious period of 8.9 days. After a person entered quarantine, they were no longer included in the analysis. The SJ church outbreak received considerable media attention beginning on August 17, 2020, after which mass testing of SJ church members and attendees was conducted. Therefore, we assumed that, after August 17, the period of onset to quarantine was 1 day, with certainty. Table 3 summarizes the parameters that were used.

### Ethics statement

Ethical approval was not required because this study was based on a series of computer simulations and did not collect any human or animal data in the study.

## RESULTS

**Figure 1.**
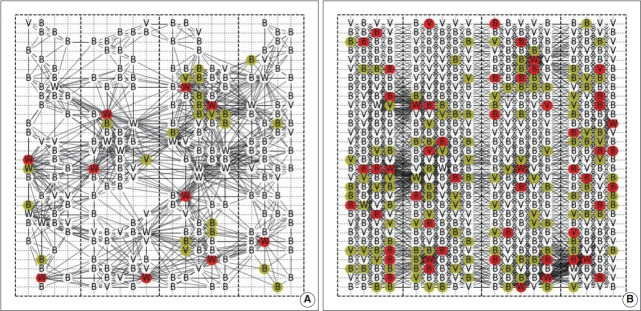
Network structure (A) weekday worship service and (B) Sunday main worship service. Uninfected people are displayed in white, infected people are in red, and people in the incubation period are in yellow. “W” for pastors and church staff, “B” for registered church members, and “V” for non-registered visitors. An edge connects two nodes if they are in a contact distance of one of those.

We assessed contact patterns and transmission dynamics of the outbreak using simulation maps of the SJ church network structure (Figure 1).

**Figure 2.**
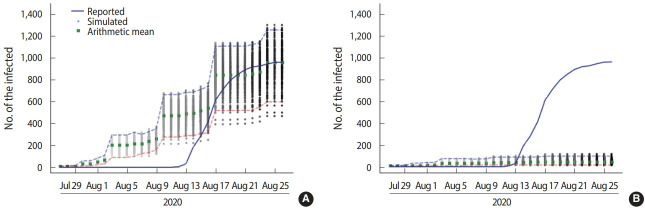
Simulation results showing daily epidemic curve (A) baseline model (mask-wearing ratio=67%) and (B) counterfactual experiment (mask-wearing ratio=95%). Blue dashed line is 99 percentile and red dashed line is 1 percentile of the simulated distribution.

Under moderate calibration, the rate of mask-wearing at the SJ church during the simulation period was estimated to be 67.0%, which closely matches actual data from the SJ church case (Figure 2A). Since there were no data available about the types of masks that were worn (e.g., cotton, surgical), only KF94 masks were considered. If non-KF94 masks were included, the mask-wearing rate would have been higher. The correct method for wearing a mask is also an important factor. Wearing a mask is not effective at protecting the wearer if it is not properly worn or if it is loosely fitted. According to media coverage of the SJ church outbreak, many of the church attendees did not strictly adhere to government guidelines, including those related to mask-wearing and social distancing. Thus, we believe the estimated rate of mask-wearing in this study was reasonable.

The baseline model with a 67.0% rate of mask-wearing estimated an average of 953.8 (95% confidence interval [CI], 937.3 to 970.4) infections with a standard deviation of 145.8. The range of the infection from the first percentile to the 99th percentile of the simulated distribution was 597 to 1,253 infections. The large increase in the rate of infection was centered around the Sunday worship gathering (Figure 2A). The difference between the simulated and confirmed numbers reflects the detection latency of COVID-19 cases.

### Counterfactual experiment: 95% mask-wearing ratio

We conducted a counterfactual experiment using a calibrated baseline model. Based on government guidelines, we estimated the size of the outbreak assuming a higher mask-wearing rate. The counterfactual experiment simulated the same incident 300 times under the assumption that 95.0% of members wore KF94 masks (vs. the baseline model of 67.0%). The 95% mask-wearing rate reflects the proportion of attendees who wore masks improperly even despite government guidelines requiring every participant to wear a mask.

The counterfactual experiment with a 95% mask-wearing rate estimated an average of 45.6 (95% CI, 43.4 to 47.9) infections with a standard deviation of 20.1. The range of the infection from the first percentile to the 99th percentile of the simulated distribution was 15 to 97 infections. Thus, if the SJ church had followed government guidelines, no large-scale outbreak would have occurred, with only about 40-50 infections in the worst-case scenario. These simulations indicate that mask-wearing could have reduced total infections by 95.2%.

### Sensitivity analysis

#### Probability of infection

**Figure 3.**
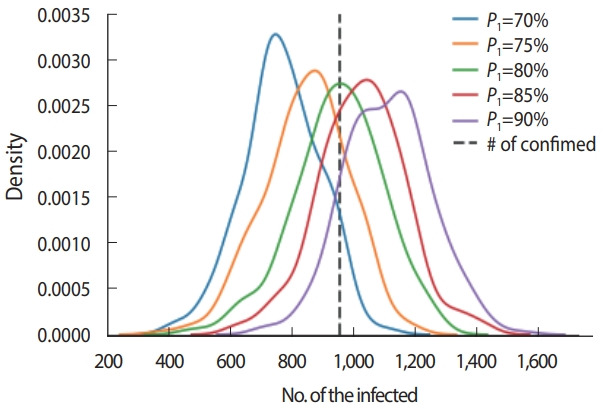
Robustness of infection probability (*P*_1_). Distributions of the estimated numbers of the cases shown when different infection *P*_1_ were applied.

The probability of infection was assumed to be 80% when infected and non-infected people wore no masks and were in contact for 1 hour (*P*_1_). We performed a sensitivity test to check the robustness of the result from 70% to 90% using 5% increments. The actual number of confirmed cases fell within all tested distributions, indicating that the probability of infection used in this study was robust within± 10% (Figure 3).

## DISCUSSION

Compartmental models have been predominantly used to understand and predict the dynamics of COVID-19 outbreaks and to assess non-pharmaceutical interventions to contain person-toperson transmission. However, these models are highly susceptible to uncertainty and often over-predict the reproduction rate and infection peak of the epidemic [21,22]. We used an SNA model to examine a COVID-19 outbreak associated with a church cluster in Seoul, Korea. We estimated that adherence to government guidelines regarding mask-wearing could have reduced the size of the outbreak by 95.2%.

Our study had several limitations. First, the SJ church was closed after the first confirmed case on August 13, 2020. In our model, we reduced the period of onset of infection to quarantine to 1 day, beginning on August 13, 2020. This may have led to an overestimation of the mask-wearing rate or an underestimation of the probability of infection. To generate the same number of confirmed cases over a shorter period, a much lower mask-wearing rate or a higher probability of infection would have been needed. Second, the practices of the SJ church are distinct from those of most churches in Korea. For example, the SJ church holds large-scale indoor worship services and gatherings with meals on weekdays. As such, the mask-wearing rate may have been overestimated since some of our assumptions were based on church practices in general. Third, due to a lack of information about the real network (e.g., the contact history of participants, the movement records of the pastors and staff, the assigned seat numbers of individuals), we were not able to use traditional metrics utilized in SNA such as centrality to analyze specific networks. Nevertheless, our study constructed a simulation model of a structured network with high randomness, which generated different network and transmission patterns during each simulation and proved that the number of the infected individuals can greatly differ according to the transmission pattern even under the same parameters. Therefore, we were able to confirm a possible range of infections that could have occurred based on a specific set of parameters. Lastly, our assumed infectious period of 8.9 days may have been longer than those used in other COVID-19 studies. A shorter transmission period would have resulted in a reduced number of cases, assuming all other variables remained the same. However, some individuals reportedly continued to attend church even after the onset of symptoms. Although our results are based on simulations and are thus subject to limitations, the precautionary implications and demonstrated value of SNA are still relevant.

Scholars are increasingly using SNA and other behavioral network-based models in studies of infectious diseases [23], and these models may also be useful for devising and evaluating social distancing measures for highly susceptible networks. This, in turn, could help address public health concerns over the social, economic, and psychological effects of extensive social distancing on individuals and society. We demonstrated the effectiveness of maskwearing by evaluating a COVID-19 outbreak using SNA. This is the first such study conducted at the micro level in the context of Korean society. SNA may be useful for evaluating the effectiveness of other government interventions to address COVID-19 in various social groups and settings. In particular, SNA can help government officials detect hotspots and further identify key actors in transmission. Simulating the transmission of COVID-19 in realworld social networks and environments will also help policymakers develop targeted or localized COVID-19 control strategies. Prioritizing such networks and individuals with targeted containment strategies will help minimize costs and significantly reduce the number of COVID-19 cases without the adverse effects of broadly implemented social distancing measures.

In conclusion, non-pharmaceutical interventions such as social distancing and mask-wearing are crucial for containing the spread of COVID-19. However, given that person-to-person contact/interaction is unavoidable in social and economic life, imposing stringent national social distancing measures in all places and organizations may incur more costs than benefits to society. It is critical to develop precise measures and guidelines for particular organizations or places that are susceptible to cluster outbreaks, such as religious organizations, nursing homes, and prisons.

## Figures and Tables

**Table 1.** Daily events of the church

Events	Day/Time	No. of participants
Daily morning prayer	Monday-Friday/5:00 a.m.	15-35
Weekday worship service 1	Wednesday/7:30 p.m.	175-275
Weekday worship service 1	Friday/8:30 p.m.	175-275
Small group gatherings	Saturday/5:00 p.m.-7:30 p.m.	205-245
Main worship		
Service 1	Sunday/7:00 a.m.	540
Service 2	Sunday/9:00 a.m.	540
Service 3	Sunday/11:00 a.m.	540

**Table 2.** Infection probability

Infected	Uninfected	Protection rate (%)	Probability of infection (*P_d_*)
Wearing a mask	Wearing a mask	100	0
	Not wearing a mask	90	0.1×*P_d_*
Not wearing a mask	Wearing a mask	60	0.4×*P_d_*
	Not wearing a mask	0	*P_d_*

**Table 3.** Parameters used in this study

Symbol	Parameter	Value
*δ_w_*	Contact distance of pastors and workers	6
*δ_b_, δ_v_*	Contact distance of members and visitors	3
*P_1_*	Probability of infection when a neighboring person is not wearing a mask	0.8
*μ_L_*	Average latent period (d)	4.2
*μ_I_*	Average infectious period (d)	8.9
